# The immunological mechanisms and therapeutic potential in drug-induced liver injury: lessons learned from acetaminophen hepatotoxicity

**DOI:** 10.1186/s13578-022-00921-4

**Published:** 2022-11-22

**Authors:** Qianhui Li, Feng Chen, Fei Wang

**Affiliations:** Division of Gastroenterology, Seventh Affiliated Hospital of Sun Yat-sen University, No.628, Zhenyuan Road, Shenzhen, 518107 China

**Keywords:** Acetaminophen, Drug-induced liver injury, Immune cell, Cytokine, Chemokine, Therapeutic strategy

## Abstract

Acute liver failure caused by drug overdose is a significant clinical problem in developed countries. Acetaminophen (APAP), a widely used analgesic and antipyretic drug, but its overdose can cause acute liver failure. In addition to APAP-induced direct hepatotoxicity, the intracellular signaling mechanisms of APAP-induced liver injury (AILI) including metabolic activation, mitochondrial oxidant stress and proinflammatory response further affect progression and severity of AILI. Liver inflammation is a result of multiple interactions of cell death molecules, immune cell-derived cytokines and chemokines, as well as damaged cell-released signals which orchestrate hepatic immune cell infiltration. The immunoregulatory interplay of these inflammatory mediators and switching of immune responses during AILI lead to different fate of liver pathology. Thus, better understanding the complex interplay of immune cell subsets in experimental models and defining their functional involvement in disease progression are essential to identify novel therapeutic targets for the treatment of AILI. Here, this present review aims to systematically elaborate on the underlying immunological mechanisms of AILI, its relevance to immune cells and their effector molecules, and briefly discuss great therapeutic potential based on inflammatory mediators.

## Introduction

Drug overdose or their metabolites is one of the leading causes of acute liver injury and is also important clinical problem and a challenge for drug development. Fundamentally, drug-induced liver injury (DILI) can be divided into three categories: predictive, idiosyncratic and indirect DILI [1, 2]. The risk of predictive DILI is related to the amount of drug-exposure and can be reproducible. The most typical example of predictive hepatotoxicity is the use of high doses of acetaminophen (APAP), which, along with aspirin, amiodarone, niacin, and methotrexate, are all direct liver damage caused by the drug itself or by its metabolites. These drugs mediate the release of DAMPs following hepatocyte injury, which are sensed by, for example, Kupffer cells (KCs), activating innate immune receptors to trigger intracellular events leading to the release of pro-inflammatory mediators, cytokines, and chemokines, and then recruit neutrophils and monocytes to the blood in a sterile inflammatory process. Unlike predictive DILI, idiosyncratic hepatotoxicity induced by certain drugs (amoxicillin clavulanate, cephalosporins, diclofenac, fluoroquinolones, isoniazid, macrolide antibiotics and nitrofurantoin) may depend on the activation of the adaptive immune system. These drugs or their metabolites covalently bind to proteins in the body to form drug/metabolite-protein adducts that can be processed by antigen-presenting cells as semi-antigens and then recognized by T cells to induce immune responses that may have a genetic or even metabolic basis, with dendritic cells acting as antigen-presenting cells (APCs) bridging the activation of innate and adaptive immunity [3]. Drugs with indirect hepatotoxic injury are usually associated with antineoplastic agents, monoclonal antibodies, risperidone, and haloperidol. Antineoplastic immune checkpoint inhibitors are associated with extensive secondary immune activation following their application [4] and tumor necrosis factor antagonists probably primarily associated with interference with humoral and cellular immunity, particularly in patients with pre-existing autoimmune disease [5]. The high reactivation rate of liver injury after re-administration of some drugs may be related to immune reconstitution after withdrawal of short-term excessive immune suppression [6]. Although the mechanisms underlying these three types of hepatotoxicity are not identical, they are not absolutely either/or, completely disconnected. The pathogenesis of predictive and idiosyncratic DILI is intrinsically linked in the pathways that trigger hepatotoxicity, and both have intrinsic immune and inflammatory responses in the body. Although there are significant differences in the extent, scope, and time limits, no matter which hepatotoxicity, it will result in a range and degree of target cell death if it reaches a certain intensity.

DILI can manifest as various acute, subacute, or chronic liver injury types, with severity ranging from asymptomatic elevation of liver enzymes to fulminant liver failure or even death [7]. Over 1100 drugs are currently known to cause hepatotoxicity, mainly including all kinds of prescription or non-prescription chemical drugs, biological agents, traditional Chinese medicine, nutraceuticals, herbals and dietary supplements (HDS) [8]. DILI is the primary cause of acute liver failure (ALF) in European and the United States, proportion up to 60% [9]. The incidence ranges from 14 per 100,000 people in France [10] to 19 per 100,000 people in Iceland [11]. In South Korea it is 12 per 100,000 inhabitants [12], while it is higher in China with an estimated incidence of 24 per 100,000 [13]. Traditional Chinese medicine or HDS (26.81%), anti-tuberculosis drugs (21.99%), and anti-tumor drugs or immunomodulators (8.34%) are the major causes of DILI in China [13]. Many drugs cause hepatotoxicity by forming reactive metabolites, which initiate cell toxicity via formation of protein adducts and trigger immune response-mediated toxicity.

APAP is a commonly used analgesic and antipyretic over-the-counter drug. Use of APAP is considered to be safe in therapeutic concentrations but can cause liver damage after an overdose, ultimately leading to ALF in severe cases [14]. APAP overdose leads to excessive generation of the highly reactive metabolite n-acetyl-p-benzoquinone imine (NAPQI), which depletes glutathione (GSH), increases oxidative stress, and causes mitochondrial dysfunction, finally triggering immune response-mediated hepatotoxicity and massive hepatocytes necrosis [15, 16]. Necrotic hepatocytes release damage-associated molecular patterns (DAMPs) that attract immune cell infiltration and initiate liver repair and regeneration [16]. It is believed that cross-linking between innate immune cells and target cells (e.g., hepatocytes), or even between innate immune cells within the liver, largely contributes to the disease process through the induction of inflammatory cytokines/chemokines [17]. In the APAP hepatotoxicity-mediated immune response, KCs form the first line of defense by recognizing necrotic hepatocytes caused by drug injury through DAMPs, and KCs, upon activation release of cytokines such as IL-6, IFN, TNF and chemokines to regulate hepatocyte function. For example, IFN can act by binding to transmembrane receptors on the surface of hepatocytes, and IFN and TNF can synergistically induce INOS expression leading to DNA breakage to induce apoptosis [18]. Meanwhile, cytokines act as mediators of neutrophils and monocytes recruitment and amplify the inflammatory process by activating the release of various inflammatory mediators from the infiltrating leukocytes. The released inflammatory mediators lead to the upregulation of adhesion molecules such as ICAM-1 on liver sinusoidal endothelial cells (LSECs) and hepatocytes as well as β2 integrins (CD11b/CD18) on neutrophils, which regulate the aggregation of immune cells through the production of mediators and the expression of adhesion molecules that assist neutrophil adhesion and transit within the sinusoids, adhesion to target cells and dependent oxidative stress, leading to hepatocyte death [19–21]. LSECs selectively inhibit Th1 cells, reducing IFN- γ production, and activate Th2 cells, leading to increased IL-4 secretion [22]. Overall, there are two possible pathways that mediate the interaction between immune cells and hepatocytes, one that may rely on direct cell-to-cell contacts such as cell surface receptors and ligand adhesion molecules, the other through, for example, inflammatory mediators released by these cells.

In the United States, APAP hepatotoxicity is the most frequent cause of ALF of any etiology, accounting for approximately 50% of all cases [23]. Up to now, only one specific pharmacological treatment option for patients suffering APAP-induced liver injury (AILI) exists: the administration of high doses of the n-acetylcysteine (NAC), which is the only FDA approved antidote for clinical use against APAP overdose. Unfortunately, the benefit of NAC administration tends to decrease with the time passed between overdose and treatment [24]. Nevertheless, due to the wide-spread use of the drug, APAP overdose is by far the most frequent cause of ALF of any etiology in many countries. Thus, the major challenges related to APAP overdose are: (1) In-depth understanding the mechanisms of toxicity and regeneration to develop novel therapeutic intervention strategies for the treatment of AILI to complement NAC in a delayed fashion; (2) Exploring predictors as early as possible after admission where patient could recover and will need a transplant to survive. Further elucidation of the cellular and molecular mechanisms of AILI can help identify the risk factors for ALF to predict prognosis as early as possible, and also facilitate the development of effective targeted therapies to prevent progression of liver injury. Although oxidative stress, mitochondrial damage, and cell death involved in the pathogenesis of APAP hepatotoxicity have been reported [25], accumulating studies on the pathogenesis of APAP hepatotoxicity indicate that subsequent inflammatory responses of the immune system critically determine the severity and outcome of disease [26, 27]. Several immune cell types, cytokines and chemokines are incorporated in the inflammatory response after acute liver injury. Based on the previous studies, there has been raising interest in understanding the underlying immunological mechanisms during AILI. Understanding the complex interplay of immune cell subsets in experimental models and defining their functional involvement in disease progression is essential to identify novel therapeutic targets for this malady. In this review, we will focus on the roles of different immune cell subsets, cytokines and chemokines in the pathogenesis of APAP hepatotoxicity and discuss the potential targets to modulate the immune response for a better clinical outcome in this malady.

## Role of immune cells in AILI

**Fig. 1:**
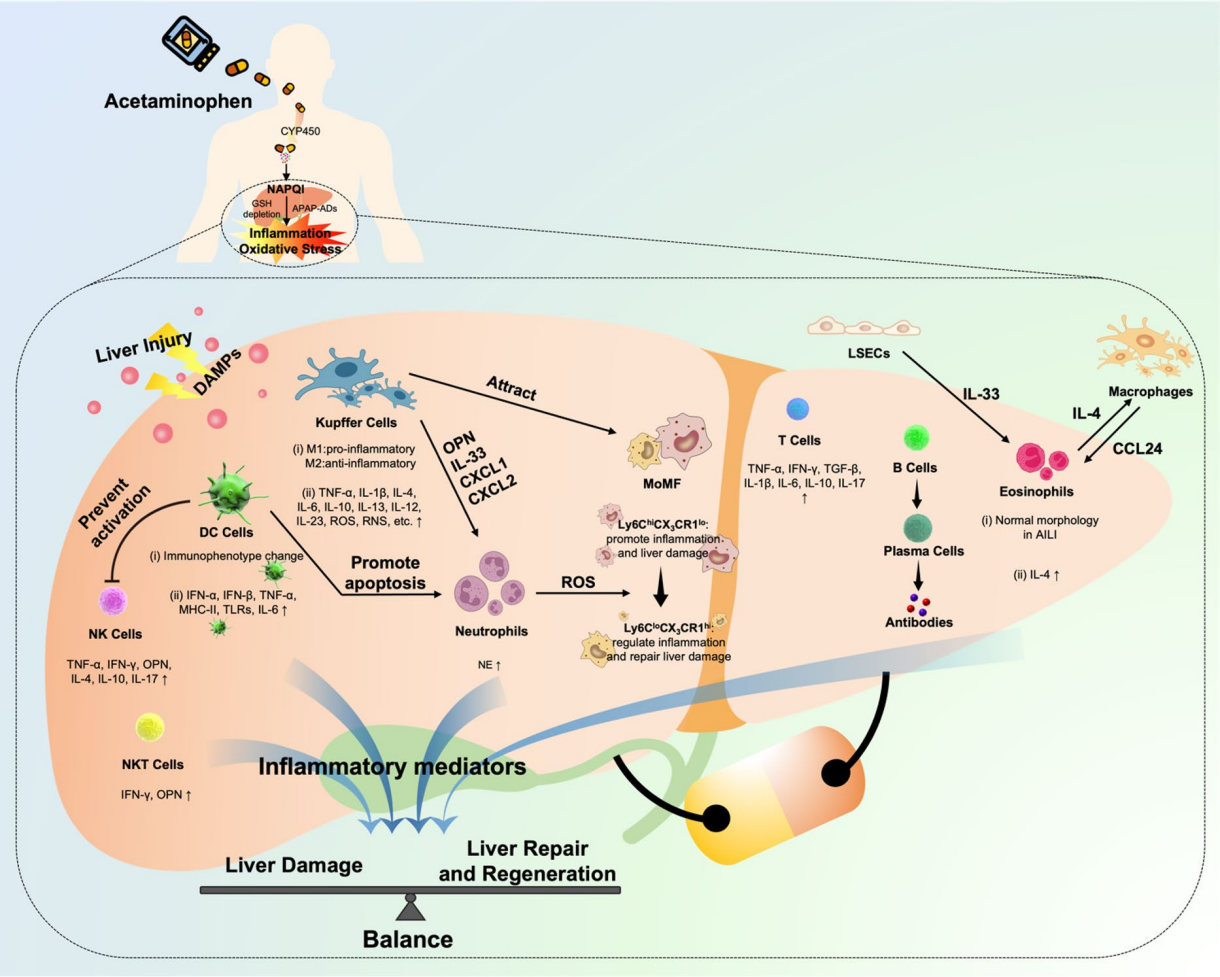
Immunologic mechanisms of AILI. Overdose APAP is metabolized by cytochrome P450 to generate the highly reactive metabolite NAPQI. Excessive NAPQI depletes GSH, leading to formation of protein adducts and acute liver injury. Necrotic hepatocytes release DAMPs that drive hepatic infiltration of immune cells. Activated immune cells secrete various cytokines and chemokines, including pro-inflammatory mediators and anti-inflammatory mediators, to modulate the balance between liver damage and liver repair and regeneration. During APAP challenge: (1) Hepatic DCs prevent NK cells activation and attract neutrophils apoptosis. (2) KCs release pro-inflammatory mediators to recruit neutrophils and attract MoMF. (3) Neutrophils release ROS to trigger the transformation of pro- inflammatory Ly6C^hi^CX3CR1^lo^ monocytes/macrophages skewing toward reparative Ly6C^lo^CX3CR1^hi^ macrophages. (4) IL- 33, selectively released by LSECs, stimulates eosinophils to secrete IL-4, which promotes macrophages to produce a plethora of eotaxin-2 (CCL24) to trigger the recruitment of eosinophils. In addition, activated B cells produce antibodies participating in AILI. CYP450 cytochrome P450, NAPQI N-acetyl-p-benzoquinone imine, GSH glutathione, APAP-ADs acetaminophen protein adducts, DAMPs damage-associated molecular patterns, NK cells natural killer cells, NKT cells natural killer T cells, DCs dendritic cells, MoMF monocyte-derived macrophages, LSECs liver sinusoidal endothelial cells,TNF-α tumor necrosis factor-α, IL-1βinterleukin-1β, ROS reactive oxygen species, RNS reactive nitrogen species, IFN-γ interferon-γ, OPN osteopontin, CXCL1 C-X-C Motif Chemokine Ligand 1, NE neutrophil elastase, MHC-II major histocompatibility complex-II,TLRs Toll-like receptors, TGF-β transforming growth factor-β

The liver is the largest organ in the human body and is responsible for the metabolism and storage of the three principal nutrients: carbohydrates, fats, and proteins. The liver also contributes to the breakdown and excretion of medicinal agents and toxic substances. In addition to its role as a metabolic center, the liver is also regarded as special immunological organ due to its enriched resident immune cell populations [28]. There are several resident immune cells in the liver, such as Kupffer cells (KCs) and some types of innate lymphocytes [29, 30]. These immune cells account for 10–20% of the total number of liver cells [31].In addition, the liver recruits circulating immune cells including macrophages, neutrophils, eosinophils, T lymphocytes, etc.[32, 33]. Immune dysfunction takes responsible to various kinds of liver diseases, including DILI, fatty liver disease, autoimmune liver disease, etc.[34, 35]. Since the complexity of liver microenvironment, the exact mechanism of immune response in the occurrence and progression of AILI remains controversial. Previous studies have shown that immune cells and inflammatory mediators play an important role in the progression of AILI [36–43]. However, numerous research indicate that immune system has a dual role in APAP-overdose challenge [36, 37, 39, 42, 43], which may be related to the specific roles of the immune cells in exacerbating liver damage or promoting liver repair and regeneration (Fig. 1).

### Kupffer cells (KCs)

Kupffer cells (KCs), also known as liver-resident macrophages, account for 80‒90% of systemic tissue macrophages and 35% of liver nonparenchymal cells, and play a central role in systemic and regional defense [44, 45]. KCs have diverse functions, including phagocytosis, endocytosis, immunomodulation and synthesis and secretion of numerous biologically active mediators [36, 39, 46, 47]. KCs have also been reported to secrete cytokines and chemokines which help recruit tother immune cells into the damage site [45]. It is believed that the regulatory role of KCs in AILI is mediated by their production of cytokines and other biologically active mediators.

Necrosis of hepatocytes causes the release of DAMPs, which can be recognized by KCs to motivate the secretion of pro-inflammatory cytokines. Previous studies have demonstrated that large number of KCs are activated and secrete TNF-α, IL-6 and IL-1β in 1 h post APAP challenge [48]. Activation of the innate immune system attracts neutrophils and monocyte-derived macrophages (MoMFs) to locate in the inflammatory site [49, 50]. In severe cases, this situation can lead to ALF and ultimately death. Evidence suggests the participation of KCs in AILI leading to the exacerbation of hepatocyte damage, however, the specific role of KCs in APAP-overdose challenge remains controversial.

It has been reported that inactivation of KCs significantly alleviates liver injury and lower transaminase levels in mice with APAP challenge [37, 42], hence demonstrating that KCs contribute to APAP hepatotoxicity. Macrophage-inducible C-type lectin (Mincle) can activate KCs through recognizing a damaged hepatocyte releasing endogenous ligand, named spliceosome-associated protein 130 (SAP130), to exacerbate APAP hepatotoxicity. KCs are the key points for the detrimental role of Mincle in AILI [51]. The main cellular source of Mincle is KCs in the liver and Mincle promotes the inflammatory response of KCs in AILI. Earlier study found that there is no statistical difference in alanine aminotransferase (ALT) levels between Mincle deficient mice and wild-type (WT) mice after APAP challenge [52]. These contrary experimental results may due to different fasting duration. KCs are considered to be associated with the alleviation of liver damage in hepatic stellate cells (HSCs) depleted mice after APAP challenge. HSCs elevate the endotoxin-stimulated expression of pro-inflammatory cytokines secreted by KCs. On the contrary, KCs do not significantly up-regulate or lower the amount of endotoxin-induced inflammatory cytokines secreted by HSCs. The possibility that the protection of HSC-depleted mice from APAP challenge may be partly related to the inadequacy of such modulated relationship between HSCs and KCs [53]. In addition to the aforementioned recently discovered evidence, KCs have been reported as promoters in AILI for releasing classical pro-inflammatory cytokines (including TNF-a, IL-1β, IL-6, IL-12, IL-23) and reactive oxygen species (ROS) and reactive nitrogen species (RNS) [42, 46, 54, 55].

Recent studies believed that KCs have a dual role in liver injury, accelerating liver damage but promoting liver regeneration [36, 37, 39, 41–43]. Earlier studies suggested that macrophages can be characterized into classical M1 type and alternative M2 type, which can interchange in response to inflammatory factors of microenvironment signals [56–58]. M1 macrophage is thought to be responsible for the promotion of liver injury caused by macrophages. They have strong microbicidal, tumoricidal, antiproliferative and cytotoxic activity. The balance between M1 and M2 type macrophages contributes to the progression of AILI [46, 57, 59]. M2 macrophage takes responsible for liver repair and controlling inflammation by releasing anti-inflammatory cytokines including IL-4, IL-10, IL-13, etc. Moreover, M2 macrophage phagocytizes apoptotic neutrophils and produces important mediators in tissue recovery [54, 60, 61]. The depletion of KCs by liposome-entrapped clodronate in mice significantly exacerbates liver damage after APAP treatment. In addition to pathogenic role, KCs also contribute to protection against AILI by producing some hepato-regulatory cytokines and mediators, including IL-6, IL-10, IL-18 binding protein and complement 1q (C1q) [42]. The supporting evidence was reported later from an American team, who demonstrated that the homeostasis and integrity of LSECs was damaged and liver injury was aggravated in KCs deficient mice after APAP treatment [62]. Another group also confirmed KCs deficiency aggravates AILI. The upregulation of multidrug resistance-associated protein 4 (Mrp4), an efflux transporter, may help to reduce the accumulation of toxic substance and aid in liver recovery, which may be one of the beneficial factors of KCs protective role in AILI [63]. Recent study showed that eliminated KCs with liposome-entrapped clodronate blocked the protective function of netrin-1 to AILI [64].

In conclusion, macrophages activation and interchange are dynamic processes: the same KCs may take responsible for inflammatory promotion and hepatotoxicity first and then help down-regulate inflammation and repair injury [54, 61].

### Neutrophils

Neutrophils are essential components of the host innate immune system. When body suffers from tissue injury, inflammation, and tumor etc., neutrophils are activated and moving to the damage site [50, 65, 66]. The activation and recruitment of neutrophils are modulated by DAMPs, other immune cells and inflammation mediators in AILI. DAMPs, including ATP, high-mobility group box1(HMGB1), release in mice after APAP challenge, induce neutrophils recruitment [67–69]. KCs activated by overdose-APAP release numerous pro-inflammatory mediators to recruit neutrophils to damage sites [45, 70]. Other study found that KCs significantly increase the secretion of osteopontin (OPN), which augments the migration and activation of neutrophils and leads to hepatocytes necrosis [71]. However, data reported from other groups are conflicting, they hold the view that macrophages may inhibit neutrophils activation [72, 73]. APAP overdose leads to the increasing expression of IL-33[74], CXCL (C–X–C Motif Chemokine Ligand)1 [75], CXCL2 [76] and other pro-inflammatory factors, finally resulting in more neutrophils activation and recruitment.

Neutrophils play an important role in the exacerbation of AILI [40]. Neutrophil elastase (NE), a secretion of activated neutrophils with cytotoxic and proinflammatory function, has been reported to be related to human disease, especially in the pulmonology [77–80]. Recent study demonstrated that the expression of NE is significantly upregulated in the liver and serum during overdose APAP challenge. Using NE inhibitors can limit the hepatic necrosis and reach a similar decrease in serum levels of ALT and AST compared with NAC-treated mice. Further study found that a combination therapy of NE and NAC has a more satisfying result than NAC monotherapy [76]. He et al. [81]*,* demonstrated that microRNA-223 (miR-233), a small non-coding RNA, makes contribution to the prevention of neutrophils overactivation to palliate AILI and knockout of miR-233 leading to an increased hepatic neutrophils infiltration to exacerbate APAP hepatotoxicity in mice. They also found that the increased expression of miR-233 in neutrophils in mice with APAP challenge is partially dependent on Toll-like receptor 9 (TLR9). Similar conclusion was confirmed by another Brazil experimental team [82]. The above evidences suggest that neutrophils play a harmful role in the pathogenesis of APAP hepatotoxicity.

In contrary, some researchers suggested that neutrophils are not involved in the early phase of APAP hepatotoxicity [69, 83, 84]. Williams et al. [83]*,* found that mice treated with extra dose of IL-1β and APAP have an increasement of neutrophils accumulation by 35% compared with APAP monotherapy, but have no significant difference in serum levels of ALT or liver necrosis. Other study claimed that neutrophils only make contribution to necrotic cell fragments removal, but not directly participate in the pathogenesis of hepatotoxicity with APAP [84].

Furthermore, some researchers proposed that neutrophils participate in the injury repair and regeneration of AILI [85–87]. Researches revealed that neutrophils contribute a lot to the transformation of pro-inflammatory Ly6C^hi^CX_3_CR1^lo^ monocytes/macrophages skewing toward reparative Ly6C^lo^CX_3_CR1^hi^ macrophages in AILI. Notably, ROS that mainly released by neutrophils may be the crucial mediator which triggers this procession [86]. Recent study demonstrated that the blockage of CLEC-2 (C-type lectin-like receptor-2) podoplanin axis reduces the hepatotoxicity and transaminase levels in AILI by increasing TNF-α related recruitment of reparative hepatic neutrophils [87].

Activated neutrophils play an important role in liver disease. Excessive infiltration of neutrophils in liver tissue may cause severe inflammation and necrosis, while phagocytosis and bactericidal to detrimental components promote tissue repair [88]. Inhibition of NE, which released by neutrophils, can effectively relieve AILI [76]. Moreover, numerous evidence supports that several compounds and pathways mitigate APAP hepatotoxicity by modulating the recruitment and activation of neutrophils, including berberine [89], geniposide [90], glycyrrhetinic acid [91], etc. [92, 93]. By clarifying the relationship between neutrophils and AILI, targeting neutrophils may become a novel promising strategy for treating APAP hepatotoxicity.

### Natural killer (NK) cells and natural killer T (NKT) cells

Liver immune system is an indispensable component of innate immune system. There is a significant distinction between hepatic resident lymphocytes and those in peripheral lymphatics [44]. In human body, NK cells account for 30–50% of liver lymphocytes [94]. Apart from innate immune response, they also make contribution to cell-mediated cytotoxicity and exocytosis of cytotoxic granules. NK cells get involved in procession of infection and tissue injury with the ability of detecting the aberrant cells [95–97]. NKT cells preferentially reside in liver, which is different from other T lymphocytes. NKT cells, which have immunomodulatory and cytotoxic function, express both T lymphocyte receptors and NK cell receptors, are the bridge between innate immunity and adaptive immunity [98, 99]. Activated NK cells and NKT cells secrete inflammatory mediators, such as TNF-α, Interferon (IFN)-γ, IL-4, IL-10 and IL-17, contributing to balance the pro-inflammatory and anti-inflammatory responses in hepatic diseases [100–104]. At present, NK cells and NKT cells have been demonstrated to play a pathogenic role in different kinds of liver diseases, including DILI, immune liver injury, liver cancer, viral hepatitis and so on [103–110].

IFN-γ, mainly secreted by activated NK cells and NKT cells, proves to be related to the release of chemokines and cytokines, promotes the infiltration of immune cells, resulting in hepatocyte apoptosis. IFN-γ-deficient mice have a reduction of transaminase levels, less area of hepatic necrosis, fewer infiltration of leukocytes, and all of them survived during overdose-APAP challenge. Thus, Ishida et al*.,* suggested that IFN-γ takes responsible for the severity of APAP hepatotoxicity by regulating leukocytes infiltration, hepatocyte apoptosis and secretion of inflammatory mediators [111]. Recent study indicated that NK cells interfere the progression of DILI, resulting in hepatotoxicity and expression of IFN-γ. Researchers analyze the primary human hepatocytes, which have been treated with 148 drugs in concentrations of clinical region, by genome-wide analysis, and find that various drugs, including promethazine, isoniazid, ketoconazole and valproic acid, can activate ligands for NK cell receptors, resulting in hepatocytes killing by NK cells [106].

NK cells and NKT cells are involved in the progression of AILI. Previous study showed that OPN in addition to being secreted by KCs, is also produced by stimulated NK and NKT cells [71, 112, 113]. OPN knockout mice have less susceptibility to AILI than WT mice [114]. Swiss Jim Lambert (SJL) mice lack of NK cells and NKT cells [115], and present lower levels of OPN in liver than B6 mice during APAP challenge [114]. Researchers conjecture the different APAP-hepatotoxicity between SJL mice and B6 mice may result from insufficient number of activated NK cells and NKT cells in SJL mice [114]. Similar protective result is obtained when using anti-NK1.1 antibody to deplete both NK cells and NKT cells in C57BL/6 mice in APAP challenge [116]. Evidence for such conclusion is the reduction of area of hepatic necrosis and transaminase levels, improvement of mice survival, downregulation of the mRNA expression of IFN-γ and chemokines. However, the relevance of these findings and conclusion has been questioned. Masson’s team [117] reported that no prevention of liver injury has been observed in mice where both NK and NKT cells are depleted, when APAP is dissolved in saline. They also found that when dimethyl sulfoxide (DMSO) given alone can activate NK cells and NKT cells in vivo, and higher release of IFN-γ is observed. Hence, DMSO should be used very carefully in the study of NK cells and NKT cells. Conflicting observations also appear in the question about the responsibility of NKT in AILI. According to Downs’s team [118], fed NKT cells-deficient mice (Jα18^−/−^mice) prevent APAP hepatotoxicity by increasing expression of GSH and changing drug metabolism. However, other study reported that NKT cells-deficient mice (DC1d^−/−^ and Jα18^−/−^ mice) show more susceptibility to AILI since starved NKT cells-deficient mice produce more ketone bodies, which up-regulate expression of CYP2E1 [105]. The results are reversed by differences in feeding conditions. Therefore, the exact role of NK cells and NKT cells in AILI still needs further investigation.

### Dendritic cells (DCs)

Dendritic cells (DCs), derived from pluripotent hematopoietic stem cells in bone marrow, are first named by Canadian scientist Ralph M. Steinman in 1973 for their distinctive dendritic morphology. DCs, as the main antigen-presenting cells in the liver, can efficiently capture, process and present antigen. They are divided into 2 subsets: myeloid dendritic cells (mDCs) and plasmacytoid dendritic cells (pDCs), make contribution to innate immunity and adaptive immunity response [119, 120]. Compared with pDCs, mDCs can express more major histocompatibility complex (MHC)-II, have stronger antigen presentation ability than pDCs. The mDCs take up antigen when being in the immature period [121] and differentiate to 2 types, just like T cell tolerance promotors or effective immunity inducers [122, 123]. As a guide for T cell differentiation, DCs integrate various signals in the microenvironment, including mediators released by inflamed, infected or injured tissues, antigens and pathogens [120]. On the contrary, pDCs concentrate on capturing viruses and act as producers of IFN-α and IFN-β [124]. They also possess the ability to present antigen to T cells, but it needs to be shown under special condition [125].

It’s well believed that innate and adaptive immunity work together during the progression of DILI, and the interaction between DCs and T cells cannot be overlooked since the damaged hepatocytes release DAMPs and toxic metabolites, which exacerbate liver injury [126, 127]. Although excess metabolites of APAP cause initial hepatotoxicity, the innate immune system of liver aggravates AILI in a form like “secondary attack”. An increasing number of evidence confirms that the severity of AILI is related to the subsequent inflammatory immune response [37, 42, 84, 111, 116, 128–131]. In chronic liver fibrosis, DCs change immunophenotype and modulate NK cells and T cells activation by TNF-α production [132]. Therefore, researchers speculated that DCs may also play a regulatory role in AILI, and proved not only the immunophenotype but also the secretion of inflammatory mediators in DCs change after APAP stimulation, including higher expression of MHC II and Toll-like receptors, and producing more IL-6, TNF-α, etc. Further studies showed that DCs depletion increases the area of liver necrosis and causes higher mortality within 48 h, while DCs amplification results in reduction hepatotoxicity of APAP [133]. Notably, although experimental team found that hepatic DCs prevent NK cells activation and attract neutrophils apoptosis during APAP challenge, the aggravation of liver injury by DCs depletion is not correlated with NK cells, neutrophils and various inflammatory mediators [133]. However, DCs depletion exacerbates liver ischemia/reperfusion injury because of a reduction of IL-10, which is a strong anti-inflammatory cytokine produced by DCs [134]. The exact role of DCs in different states of acute liver injury is undefined, and it may be the key point to figure out the relationship between hepatic immune response and liver injury.

### Eosinophils

The number of eosinophils shows a circadian rhythm in blood, normally with less cells in the early morning, which is considered to be mainly related to the diurnal fluctuation of the levels of glucocorticoids. Eosinophils, which secrete cytokines and enzymes to eliminate pathogens or host cells, have long been thought to participate in the process of allergic disease and parasitic infection [135, 136]. By further study, scientists found that eosinophils are also involved in drug-induced diseases. In the past 20 years, several cases of APAP associated with pulmonary eosinophilia have been reported [137]. Eosinophils are also related to DILI, including APAP, enalapril, carbamazepine, etc.[33, 138–140] Eosinophils recruitment in patients with DILI correlate with the levels of eotaxin, a potent eosinophil chemoattractant [141, 142]. Eosinophils remain morphologically normal in APAP hepatotoxicity, whereas degranulated or lytic eosinophils are present in hepatotoxicity induced by other hepatotoxin (e.g., penicillamine, ketoconazole, halothane, etc.) [140].

Emerging evidence suggests that eosinophils contribute to promoting damaged tissue repair and reducing inflammation. Eosinophils against acute lung injury [143] and relieve airway inflammation [144]. Scientists unravel the protection of eosinophils in liver injury. Patients who with peripheral and hepatic eosinophilia in DILI are more likely to have lower bilirubin levels and generally gain a favorable prognosis [145]. Eosinophils could promote liver regeneration after partial hepatectomy or carbon tetrachloride (CCl_4_)-induced liver injury, due to the secretion of IL-4 which can promote hepatocyte proliferation. Mice with liver eosinophil absence, result in impaired regenerative response [146]. Eosinophils recruitment and protective role are common in numerous models of acute liver injury, including AILI. In overdose-APAP challenge, IL-33, selectively released by LSECs, stimulates eosinophils to secrete IL-4, which promotes macrophages to produce a plethora of eotaxin-2 (CCL24) to trigger the recruitment of eosinophils [33]. These findings make eosinophils a promising cell-based therapy for APAP hepatotoxicity.

Despite increasing evidence supports eosinophils protect against AILI, there is no denying that eosinophils have a dual role of both aggravating liver injury and promoting liver repair in DILI, and this may depend on the type of drugs. For example, eosinophils play a pathogenic role in the mouse model of halothane-induced liver injury (HILI). During the early phase of HILI, eosinophils infiltrate in the liver injury sites, and the amount of eosinophils increase proportionally with the severity of damage. Moreover, a reduction of halothane hepatotoxicity is found in mice with absence of eosinophils [147]. In general, studies implied that the specific role of eosinophils in DILI depends on the type of drugs, and there is more evidence supporting protective mechanism for APAP hepatotoxicity. The possible positive role of eosinophils in the prevention and treatment of AILI deserves further investigations.

### T lymphocytes (T cells)

T lymphocytes, the major players in adaptive immunity, mature in the thymus and migrate to immune organs and damaged tissues through the lymph circulation or blood circulation [148]. There is a large amount of T cells, which have different phenotypes with circulating lymphocytes, resident in healthy portal tracts and liver parenchyma [149]. According to their surface markers, T cells are divided into CD4^+^ helper T cells (Th), which can assist humoral immunity and cellular immunity, and CD8^+^ cytotoxic T cells (Tc), which can kill target cells. Based on the cytokines secretion, Th cells can be further divided into 4 subtypes, including Th1, Th2, Th17 and regulatory/suppressor T cells (Treg). Also, there is a small amount of non-classical T cells in the liver, called γδ-T cells [150]. Th1 cells secrete IFN-γ, Th2 cells mainly produce IL-4, IL-5 and IL-13, while activated γδ-T cells can rapidly release IL-17 and IFN-γ to regulate the immune response [150–152]. Activated T cells participate in immune regulation by releasing inflammatory mediators (e.g., cytokines, chemokines) and cytolytic mediators. T cells not only contribute to maintaining liver tolerance, but are also important participants in liver damage and inflammation [153–155].

The balance of cytokines secreted by Th1/Th2 cells is critical for AILI progression. Different strains of mice were used in the experiment, the liver damage of C57BL/6 (Th1 dominant) mice after intraperitoneal administration of APAP was more serious than that of BALB/c (Th2 dominant) mice. This difference is mainly due to the release of cytokines. A plethora of TNF-α is observed to be released from C57BL/6 mice with APAP challenge, while BALB/c mice express higher levels of IL-6. Thus, mice with Th1 cells as the main response are more susceptible to AILI, resulting from producing more TNF-α [156]. An increasing number of DC64L^LOW^DC44^HI^CD4^+^ T cells is observed in the liver after APAP treatment, accompanied with higher secretion of IFN-γ. CD4^+^ T cells depletion alleviate liver injury induced by APAP [157]. Treg cells have also been found to recruit to liver by CXCL10-C-X-C chemokine receptor (CXCR)3 axis and secrete anti-inflammatory mediators IL-10 and transforming growth factor (TGF)-β to ameliorate AILI [157]. Therefore, regulating the balance between Th1/Treg cells would be a promising strategy to treat AILI. Meanwhile, the complex interrelationship between Th1, Th2 and Treg cells in AILI treatment needs more attention and investigation.

Th17 cells are also involved in the pathogenesis of AILI. The number of Th17 cells increases within 6 h after APAP challenge and releases IL-17, which modulates inflammation by promoting the secretion of pro-inflammatory factors and neutrophil-mobilizing cytokines [158], IL-17 deficiency reduce AILI in mice [159]. However, the response of Th17 cells is too fast for adaptive immune cells, leading to a speculation that Th17 cells may be part of innate immunity. TNF-α and IL-1β are released rapidly by KCs after APAP treatment [48]. A study on endometriotic stromal cells showed that CCL20/CCR6 axis attracts selective migration of Th17 cells, and TNF-α as well as IL-1β induced the secretion of CCL20 [160]. In recent years, certain type of cells, which breaks down the boundary between innate and adaptive immunity, have come into sight. Innate lymphoid cells (ILCs), belonging to the innate immune system, exhibit part of the function that be previously considered as unique to adaptive immune cells [161]. More and more evidence supports that there are doppelgangers of each type of helper T cells in the innate immune system [162, 163]. These findings may partly explain the mechanism of Th17 cells rapid response after APAP challenge.

In addition to Th17 cells, IL-17 can be released by various kinds of cells, including γδ-T cells [164, 165]. Depletion of γδ-T cells decreases IL-17 production and APAP hepatotoxicity [67]. Baicalin (BA), extracted from herb medicine called radix scutellariae, decreases the recruitment of γδ-T cells after APAP administration, and lower the expression of IL-17 to alleviate liver damage [166]. The HMGB1-TLR4-IL-23 axis of macrophages increases the secretion of IL-23 to promote γδ-T cells to produce IL-17, which stimulates neutrophils infiltration and aggravate APAP hepatotoxicity [67].

CD8^+^ cytotoxic T cells are observed to infiltrate in acute liver injury, including DILI, autoimmune hepatitis and viral hepatitis [167, 168]. Similar evidence is found in patients with floxacillin-induced liver injury [169]. Numerous case–control studies of genetic susceptibility to DILI have been reported. One hypothesis for genetic susceptibility of human leukocyte antigen (HLA) is related to the response of T cells to drug, metabolite or adduct with protein [170].

B lymphocytes also play an important role in adaptive immunity. Activated B cells become plasma cells and secrete antibodies involving in humoral immunity. Although antibodies have been observed in some studies on DILI [171, 172], they only indicate DILI is associated with humoral immunity, the mechanism of B cells in DILI remains undefined.

## Role of inflammatory mediators in AILI

Cytokines, which have various functions including immune regulation, intercellular signal transmission, and damaged tissue repair, etc., are small molecule proteins produced by immune cells and some non-immune cells. This effector immune mechanism involving cytokines has received extensive attention in APAP-induced hepatotoxicity, in which DAMPs released by damaged hepatocytes act on pattern recognition receptors (PRRs) on macrophages and other potential cells, causing the production of pro-inflammatory cytokines and chemokines, thus promoting the inflammatory response [173]. The severity of liver lesions depends on the involvement of subsequent inflammatory mediators and immune cells [70, 116]. Evidence suggests that cytokines involved in inflammatory induction and the immune response are one of the most important mechanisms for the occurrence and development of AILI. Also, these inflammatory mediators may aggravate the immune response and lead to severe liver injury, or some inflammatory mediators can promote hepatocytes regeneration and alleviate APAP-induced hepatotoxicity. Therefore, we focus here on summarizing the current status and application value of inflammatory mediators such as cytokines, chemokines, and inflammasomes in AILI, to provide valuable reference information for the early diagnosis and prognosis of AILI.

### Cytokines

#### IL-1 family

The IL-1 family plays an important role in immune regulation and inflammatory processes, and the roles of its members IL-1α, IL-1β, IL-1 receptor (IL-1R) antagonists, IL-18, IL-33, and IL-36 in APAP-induced hepatotoxicity have been partially elucidated. Studies show that APAP promotes the production of IL-1α and IL-1β, and more kinds of literature report on IL-1β, which plays a key role in the development of AILI, and the decrease of IL-1β alleviates liver injury caused by APAP [174–178]. IL-1R has been found to play an essential role in promoting inflammatory response and subsequent pathogenesis after APAP overdose. IL-1R deficient mice are almost completely resistant to AILI [179], and the use of recombinant human IL-1R antagonists also has a protective effect on AILI [55]. However, some studies suggest that IL-1R could not directly cause cell death, so the primary mechanism of IL-1β affecting AILI may be the activation of inflammatory cells, such as neutrophils [83]. Benzyl alcohol protects AILI by blocking the release of plasma IL-1β and IL-18 through a TLR4-dependent mechanism, and TLR4 knockdown results in the disappearance of the protective effect of benzyl alcohol [180]. Blocking the biological activity of IL-18 in mice by IL-18 binding protein (a natural antagonist of IL-18), can ameliorate APAP-induced acute liver injury [181].

IL-33, also known as IL1F11, was first identified as a nuclear factor existing in human lymph node endothelial cells [182]. A large amount of IL-33 is released during APAP challenge, and IL-33 deficiency motivates hepatocyte autophagy and interrupts M2 macrophage polarization to exacerbate liver injury, and recombinant IL-33 can reverse this phenotype [183]. IL-33 promotes macrophages to produce CCL24 by stimulating eosinophils to release IL-4, and IL-33 deficient mice exhibit impaired eosinophils recruitments, which aggravates APAP-induced inflammatory response [33]. In another study, blocking the IL-33/IL1RL1 axis could activate liver-resident infiltrating non-parenchymal cells and inhibit the release of chemokines CXCL1 and CXCL2, thereby attenuating APAP-mediated organ injury [74]. In addition, blockade of IL-36γ by IL-36 receptor antagonist reduces CCL20 levels in mouse liver, but increases tissue damage parameters, thus exacerbating AILI [184].

#### IL-2 family (γc family)

There are five members of the IL-2 family, including IL-2, IL-4, IL-13, IL-15, and IL-21, whose signaling transduction depends on the γc chain. Some studies have shown that APAP overdose promotes the production of IL-2, IL-4, and IL-21, but there is a controversy regarding the role of IL-4 in APAP hepatotoxicity [185–188]. Some studies concluded that IL-4 plays a pathogenic role in the development of APAP-induced hepatotoxicity [185], but other studies have shown that transgenic C57BL/6 mice lacking IL-4 or IL-13 have increased sensitivity to AILI [187, 188]. Yee et al. found that IL-13 is a critical hepatoprotective factor in AILI and endogenous IL-13 protects the liver against AILI by down-regulating the liver toxins including neutrophils, cytotoxic NK and NKT cells, cytokines, and chemokines [187]. IL-15 is a multifunctional cytokine produced by various cells. IL-15 regulates the adaptive immune system and influences the development and function of innate immune cells [189]. IL-15 knockout mice with APAP overdose increase the infiltration of inflammatory cells and production of inflammatory cytokines, including IL-1β, TNF-α, IL-6, intercellular adhesion molecule-1 (ICAM-1), vascular cell adhesion molecule-1 (VCAM-1), macrophage inflammatory protein (MIP) -1α, MIP-2α, leading to an overactive inflammatory response and enhances susceptibility to AILI [190].

#### IL-12 family/IL-6 family

This family consists of five members, including IL-6, IL-12, IL-23, IL-27 (IL-30), and IL-35. IL-6 is a multipotent cytokine with a wide range of functions, which can regulate immune response, acute phase response, hematopoiesis, growth, and differentiation of various cells. It plays a crucial role in the body’s immune response against infection. Multiple data show that the clinical outcome of APAP-induced ALF is severely affected by the initial damage to hepatocytes and the post-injury inflammatory response. IL-6 is one of the most widely used pro-inflammatory cytokines in the study of APAP hepatotoxicity. Plentiful clinical and experimental animal data indicate that IL-6 is highly induced in AILI and inhibiting IL-6 expression by multiple interventions can significantly alleviate the liver injury caused by APAP. All these studies suggest that IL-6 plays a considerable role in promoting the inflammatory response process in AILI [72, 175, 177, 191, 192]. However, a few contrary reports suggest that IL-6 deficient mice lacking the expression of cytoprotective heat shock proteins (HSPs) would be more susceptible to APAP hepatotoxicity [193]. These conflicting findings are challenging to interpret and may be related to many factors, including different animal models, different effects of reagents, or genetic interventions on inflammation. Some studies suggest that inflammation contributes to liver injury in the early stage of AILI and promotes liver regeneration in the late stage [131].

APAP induces an elevation in IL-12, which can be reversed by decreasing nuclear factor (NF)-κB p65 and upregulating IκB (inhibitor of NF-κB) -α expression by silkworm pupa oil [194], and aloe vera treatment can also reduce the number of IL-12 positive staining cells in APAP liver injury tissues [195]. There is a close association between IL-23 and IL-17A in AILI, APAP induced injury-related liver inflammation via the HMGB-1-TLR4-IL-23-IL-17A axis, in which IL-23 produced by HMGB-1-TLR4-mediated macrophages is required for IL-17A production by γσ T cells [67]. A study by Abdelaziz et al. suggests that the potential protective mechanism of celastrol and brilliant blue G combination against APAP hepatotoxicity is partly attributed to the reversal of dysregulated production of pro-inflammatory factors, including IL-17A, IL-23, and TNF-α [196]. To date, we have not found any literature reports that IL-27 and IL-35 are associated with APAP-induced hepatotoxicity, which may deserve exploration.

#### IL-10 family

**Fig. 2:**
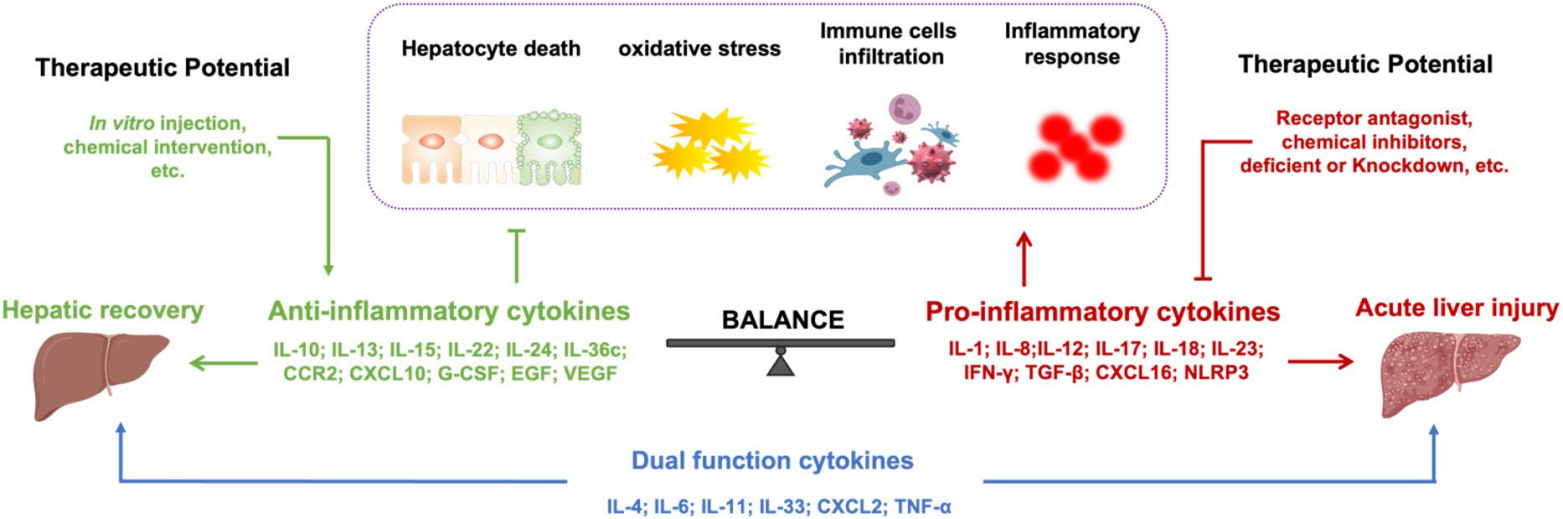
The balance of cytokines determines the outcome of the liver. Summarizing the results elaborated in the article, it was found that the green part is anti-inflammatory cytokines and the red part is pro-inflammatory cytokines, and the cytokines located in the middle have dual functions in AILI. Pro-inflammatory cytokines accelerate liver injury by promoting hepatocyte death, neutrophil infiltration, and inflammatory response. Thus, administration of receptor antagonists and chemical inhibitors reducing the expression of pro-inflammatory cytokines or in vitro injection and chemical intervention elevating the expression of anti-inflammatory cytokines may promote hepatic recovery. IL interleukin, CCR2 C–C motif chemokine receptor 2, CXCL C-X-C Motif Chemokine Ligand, G-CSF granulocyte-colony stimulating factor, EGF endothelial growth factor, VEGF vascular endothelial growth factor, IFN-γ interferon-γ, TGF-β transforming growth factor-β, TNF-α tumor necrosis factor-α

The IL-10 family is a subfamily of class II cytokines that exert various regulatory effects on the immune system, with family members including IL-10, IL-19, IL-20, IL-22, IL-24, IL-26, IL-28, and IL-29. It is known that the balance between pro- and anti-inflammatory cytokines produced in the liver determines the development of APAP hepatotoxicity (Fig. 2). Studies have shown that APAP promotes IL-10 production in the liver, and IL-10 deficient mice have increased sensitivity to AILI [197]. IL-10 may be involved in inhibiting the production of pro-inflammatory cytokines, and inducible nitric oxide synthesis (iNOS), knockdown of IL-10 exacerbates the extent of liver injury [198]. Methane-rich saline increases IL-10 levels while decreasing TNF-α and IL-6 levels through inhibiting NF-κB-mediated pathways, which has a protective effect against APAP-induced liver inflammation [199]. Suppressor of cytokine signaling (SOCS) 2, a regulatory cytokine and growth factor signaling in hepatocytes, can inhibit AILI by regulating pro-oxidative and inflammation-related mechanisms. APAP overdose increases the release of pro-inflammatory cytokines, reduces IL-10 production, and promotes neutrophil recruitment to enlarge the lesion site in SOCS2 knockout mice [200].

IL-22, a significant member of the IL-10 family, is secreted by innate lymphocytes and activated helper T cells. IL-22 is a dual nature cytokine with context-dependent protective and pathogenic properties during tissue injury. In vitro prophylactic injection of IL-22 can significantly reduce the expression of pro-inflammatory factors IL-6, IL-1β, TNF-α, and the liver necrosis area and increase the expression of hepatocyte proliferation marker Ki-67, suggesting that IL-22 through promoting hepatocyte proliferation mediates hepatoprotective functions and has the therapeutic potential in AILI [174]. IL-22 downregulates NOD-like receptor thermal protein domain associated protein 3 (NLRP3) inflammasome activation and mature IL-1β release in APAP-related injury tissue. It significantly improves the inflammatory response by effectively inhibiting pro-inflammatory cytokine IL-18, TNF-α, IL-6, and IL-1β levels [201]. IL-22 binding protein (IL-22BP) inhibits IL-22 activity. In the model of acute liver damage induced by APAP, IL-22BP deficient mice increase the infiltration of inflammatory CD11b^+^Ly6C^+^ monocytes in the liver and increase CXCL10 expression. Neutralization of CXCL10 reverses the disease susceptibility in IL-22BP deficient mice, suggesting that IL-22BP plays a protective role in AILI by regulating IL-22 signaling [202]. IL-22 pretreatment significantly upregulates hepatic LC3-II(the PE-conjugated form of light chain 3) and phosphorylation of AMP-activated kinase (p-AMPK) in APAP-treated mice, and IL-22-mediated LC3-II conversion and protection against APAP-induced cytotoxicity are impaired when p-AMPK is blocked by compound C (an AMPK inhibitor) [203]. IL-22 tethered to apolipoprotein A-I targets and alleviates AILI, suggesting that the IL-22-targeted delivery strategy may be broadly used in protecting against hepatocyte injury [204]. A recent study shows that IL-22 expression is significantly increased in the liver of female mice with APAP-induced hepatotoxicity, indicating the necessity to consider gender-dependent effects when using mice to establish a pathophysiological model of IL-22 [205]. Disruption of IL-24 increases cell death in APAP-stimulated mouse hepatocytes, significantly decreased levels of IL-24 in patients with clinical ALF may be associated with disease progression, thus IL-24 is a potential biological indicator of prognosis or therapeutic intervention in patients with liver injury [206]. The role of the remaining members of this family in AILI has not been reported.

#### IL-17 family

IL-17 family cytokines have only two interleukin members, IL-17 and IL-25 (IL17E). IL-17 family cytokines are two-fold, as they can induce cells to secrete active molecules to promote the body’s resistance to infection. However, they accelerate the course of many chronic diseases in some cases. Lee et al. found that IL-17 exerts its damaging effect through the recruitment of inflammatory cells [159]. Knockdown of IL-17 significantly reduces ALT levels, decreases hepatic neutrophils infiltration and the area of hepatic necrosis, and inhibits the production of pro-inflammatory factors TNF-α, IL-6, and IFN-γ, thereby attenuating APAP-induced hepatotoxicity [159]. IL-17 is consistently expressed during the early initiation and effector phases of the immune response during APAP hepatotoxicity, suggesting that intrinsic immune cells such as invariant NKT cells or currently unidentified macrophages may be the source of IL-17 [158]. Baicalin alleviates AILI by decreasing IL-17 levels and suppressing the recruitment of IL-17-producing γδ T cells in the liver [166]. In addition, capmatinib also alleviates AILI by reducing the overproduction and release of the pro-inflammatory mediator IL-17A [207], all of which suggests that IL-17 may play a pathogenic role in APAP-induced hepatotoxicity. The content related to IL-17A is also described in the IL-12/IL-6 family section. At present, no literature has reported the functional role of IL-25 in AILI.

#### Other interleukin cytokines

In this section, we will focus on the role of IL-5, IL-7, IL-9, and IL-11 in AILI, the role of other unclassified interleukin cytokines such as IL-14, IL-16, IL-31, and IL-32 in AILI has not been reported. IL-5 is a Th2 cytokine that has been confirmed to be associated with increased liver injury in hepatitis induced by lipopolysaccharide and concanavalin A. Only a few articles reported that APAP increases IL-5 expression in mouse liver [208, 209]. Ginsenoside Rg3 reduces some inflammatory factors, including IL-5, and alleviates APAP hepatotoxicity [208]. De León-Nava et al. do not find leukocytes infiltration in the liver parenchyma during APAP-induced ALF, probably because IL-5 causes liver injury in a leukocyte-independent manner or that IL-5 expression is an indirect consequence of liver injury and has no effect on pathology [209]. Researchers examined the expression of 40 cytokines in APAP-induced hepatotoxicity model mice and showed that APAP increases tenfold levels of IL-1α, IL-7, IL-17, CCL1, CCL3, CCL4, macrophage colony-stimulating factor (M-CSF) and CXCL9. Thiacremonone pretreatment significantly decreases the levels of the above cytokines and increases the expression of IL-1RA (interleukin-1 receptor antagonist), CXCL2, and CXCL10, thus alleviating AILI [210]. Immunological analysis of serum samples from patients with AILI reveals that they express higher levels of IL-6 and MCP-1, but lower levels of IL-9 [211]. APAP highly induces IL-11 secretion by hepatocytes, and IL-11 deficient mice exhibit spontaneous liver repair [212], however some studies demonstrate that IL-11 expression has compensatory and cytoprotective effects [213]. Omega‑3 polyunsaturated fatty acids exacerbate AILI by modulating extracellular regulated protein kinases (ERK)1/2-mediated Fra 1 expression to inhibit the production of IL-11 and further activate downstream signal transducer and activator of transcription 3 (STAT3) in hepatocytes [214]. However, a recent study suggested that upregulated IL-11 drives APAP-induced hepatocyte death, and specific deleting IL-11RA1 in hepatocytes or IL-11 knockdown in mice protected against AILI [215]. Fox et al. examined IL-11RA expression in HepG2 and primary rat hepatocytes (PRHs) after being exposure to high doses of APAP. The results showed that IL-11RA mRNA expression is significantly upregulated in HepG2 cells, but decreases to half of what in control in PRHs, upregulation of IL-11RA may be a protective mechanism against APAP toxicity and associated oxidative stress [216]. Recombinant human IL-11 (rhIL-11) is shown to be protective in AILI, rhIL-11 directly affects hepatocyte gene expression, and macrophage-mediated production of the pro-inflammatory factor TNF-α is reduced by 40–50% [217]. Moreover, providing recombinant STAT3-activated cytokines that directly target hepatocytes, specifically IL-11 and IL-22, may be another novel pro-regenerative therapeutic option for patients with difficult-to-treat APAP-induced ALF [218].

#### Tumor necrosis factor

TNF-α is a pro-inflammatory cytokine that kills target cells, promotes apoptosis, and activates the inflammatory cascade response. Studies have shown that TNF-α is pleiotropic in the development of AILI. The role of TNF-α in hepatotoxicity is related to other cytokines networks, and the interactions between different cytokine cascades determine the nature of the inflammatory response associated with toxicity. TNF-α acts synergistically with other early inflammatory mediators to promote early liver injury [131]. APAP-induced hepatotoxicity significantly upregulates TNF-α expression, but the role of TNF-α in AILI remains controversial. TNF-α or TNF-R1 knockout animals have reduced liver injury after APAP administration, suggesting that TNF-α exerts a toxic effect [219–223]. TNF-R1 deficient mice are resistant to AILI, which is associated with reduced inflammatory cytokines, chemokines, and hepatic inflammatory cell accumulation [219]. However, the role of TNF-α in the liver is very complex, on the one hand, high doses of TNF-α lead to hepatocyte death, on the other hand, it is an essential factor affecting liver regeneration and hepatocyte proliferation. Laverty et al. believed that the relationship between TNF-α and AILI may have a role in liver repair and defense against toxicity [224]. TNF-α can make quiescent hepatocytes more sensitive to growth factors, early elevation of TNF-α during APAP toxicity may be an important factor in stimulating hepatocytes and promoting late liver regeneration [225].

Several intervention studies confirmed that TNF expression alters the severity of AILI [89, 166, 191, 226, 227]. Combination therapy of boldine and NAC significantly reduces the expression of inflammatory markers, including TNF-α, IL-1β, and IL-6 [228]. Poria cocos polysaccharides pretreatment attenuates AILI by decreasing the expression of TNF-β and tumor necrosis factor receptor (TNFsR)-I [229]. Soluble death receptor 5-Fc fusion protein (sDR5-Fc) reduces AILI and leukocyte infiltration by blocking TNF-related apoptosis-inducing ligand (TRAIL) and downregulating the levels of inflammatory cytokines TNF-α, IL-6, IFN-γ, IL-4, IL-2, and IL-1β [230]. Deletion of TNF-α or TNF-R1 inhibits the positive effect of intestine-derived lipopolysaccharide to AILI, suggesting that the TNF-α/TNF-R1 pathway is required for protection against AILI [231]. In a recent study, capmatinib decreases APAP-induced elevation of TNF-α as an important mechanism to limit liver injury, as exposure to exogenous recombinant TNF-α increases APAP-induced serum ALT by more than 50%, whereas IL-1β or IL-6 increases ALT by only 20% [207]. In addition, excessive APAP upregulates TNF-α, TGF-β, IL-6 and platelet-derived growth factor (PDGF) -B levels in liver tissues of ecto-nucleotide triphosphate diphosphohydrolase-2 (NTPDase2) deficient mice, thereby aggravating AILI and increasing hepatic necrosis [232]. In conclusion, further studies are needed to confirm the specific and exact role of TNF in AILI.

#### Interferon

IFN has broad-spectrum antiviral, antitumor and immunomodulatory functions and is involved in various liver injury pathological processes. Numerous studies have shown that AILI highly induces intrahepatic IFN-γ expression and IFN-γ is involved in AILI by regulating macrophages activity, leukocyte infiltration, hepatocyte apoptosis, and the production of nitric oxide, cytokine and chemokine [57, 111]. A study based on M1/M2-macrophage function in relation to DAMPs and autophagy shows that M1 macrophages and their associated factors, including IFN-γ, MCP-1, IL-1β, IL-6, and TNF-α, are increased in the early stages of APAP-induced hepatotoxicity and are involved in tissue damage or inflammation, while M2 macrophages and related factors IL-4, IL-10, and TGF-1 subsequently appear in the late stages [57]. IFN-γ-deficient mice have lower gene expressions of molecules including ICAM-1, VCAM-1, IL-1α, IL-1β, IL-6, TNF-α, MCP-1 and iNOS after APAP administration compared with WT mice, and reduce disease susceptibility to APAP [111]. 5-lipoxygenase knockout mice reduce the production of hepatic cytokine IL-1β, TNF-α, IFN-γ, and IL-10 in APAP challenge [233], and MIF knockout mice decrease IFN-γ production and increase HSPs expression [234], all these knockout mice attenuate AILI by decreasing IFN-γ production. Studies have shown that IFN-γ-inducible protein 10 (CXCL10) exerts hepatoprotective effects in AILI by inducing CXCR2 upregulation in hepatocytes [235]. Overdose APAP stimulates CD62L^low^CD44^hi^CD4^+^ T cells infiltration of the liver, accompanied by elevated IFN-γ. Removal of CD4^+^ T cells by antibody deficiency or genetic defects reduces the levels of IFN-γ and TNF-α, and alleviates liver injury. Moreover, the recruitment of Treg cells into the liver by specific expression of CXCL10 can also ameliorate AILI [157]. Liu et al. suggested that NK and NKT cells play a vital role in the severity and progression of AILI by producing IFN-γ and regulating chemokines production and recruitment of neutrophils into the liver [116]. Overall, the immune neutralizing effect of IFN-γ has a therapeutic potency in AILI. Treated with anti-IFN-γ antibody 2 or 8 h after APAP overdose significantly attenuates AILI in mice [111].

Knockdown of interferon α/β receptor (IFNAR^−/−^) mice inhibits the IFN-1 sensing pathway. It delays AILI, and pathways associated with type I IFN production (Ifnb and Ifna4) are specifically upregulated in liver non-parenchymal cells [236]. Another study showed that an impaired ability to express iNOS in type I IFN receptor-deficient mice are associated with AILI reduction [237]. HSCs play a crucial role in AILI through the IFN-β-interferon regulatory factor-1 (IRF-1) signaling pathway, and anti-IFN-β antibodies inhibit APAP-induced hepatocyte apoptosis [53]. Infusion of activated HSCs-derived paracrine factor (HSC-CM) in the AILI mouse model significantly reduces the expression of IFN-γ, IL-1RA, IL-1β, TNF-α and leukocytes infiltration, enhancing the hepatoprotective response in mice [178]. When APAP is combined with IFN-β, it significantly altered the transcription profile of hepatocytes, including the upregulation of genes associated with cell differentiation and the reduction of interferon-inducible genes (*Ifit-3*, *Isg-15*, *Oasl1*, and *Zbp1*), indicating that there may be complex interactions between APAP and IFN-β [238]. A recent study found that emodin protects the liver against AILI by inhibiting the expression of IFN-α, cyclic GMP-AMP synthase (cGAS), and its downstream signaling effector stimulator of interferon genes (STING) [239]. In conclusion, these studies suggest that a protective effect can be provided to the liver by regulating the production of interferon.

#### Colony stimulating factor

Colony stimulating factor (CSF) makes a contribution to the non-specific cellular immune process against infection. Evidence suggests that CSF is also involved in AILI. Gao et al. found a reduction of granulocyte-CSF (G-CSF) and granulocyte–macrophage-CSF (GM-CSF) in AILI [208], while Viswanathan et al. found a higher abundance of GM-CSF and G-CSF expression in conditioned medium of AILI-derived hepatocytes [186]. In the plasma of an 18-year-old patient with APAP-induced ALF, an increased expression of IL-6, IL-8 and G-CSF is found, and elevated G-CSF levels may contribute to hepatic neutrophil vacuolization [240]. Numerous studies have shown that G-CSF can be used as a protective agent for AILI [241–243]. The efficacy of G-CSF is comparable to NAC due to it attenuates hepatic enzyme profiles and oxidative stress parameters and ameliorates APAP-induced liver inflammation and hepatocyte necrosis [241]. G-CSF significantly increases phagocytosis and killing capacity of neutrophils that are reduced in patients with APAP-induced ALF [242]. Excessive APAP-treated human hepatocytes and PRHs express G-CSF receptor (G-CSFR) and GM-CSF receptor (GM-CSFR) involved in JAK/STAT3 signaling [243]. Livers from APAP hepatotoxic mice have the defective expression of G-CSF and vascular endothelial growth factor (VEGF) [186], and replacement of the expression of these factors or receptors by transplantation of healthy hepatocytes would further improve liver homeostasis and promote liver regeneration. Mice lacking G-CSF weaken the protective role of neutrophils in liver repair during APAP challenge [86]. APAP overdose enhances the inflammatory response in serine protease inhibitor family B (SERPINB)3 transgenic mice, which may be related to the expression of higher levels of GM-CSF in transgenic mice [244].

#### Growth factor

This section focuses on the role of TGF, hepatocyte growth factor (HGF), epidermal growth factor (EGF), PDGF and VEGF in APAP-induced hepatotoxicity. TGF-β1 is a multifunctional cytokine that plays a vital role in a variety of cellular processes, including cell proliferation, growth inhibition and cell death. APAP highly induces the expression of TGF-β1 and its downstream signaling in hepatocyte necrotic regions, and blocking TGF-β1 signaling by TGFβ receptor 1 inhibitor (GW788388) ameliorates hepatocyte inflammation and hepatocyte injury [245]. A study analyzing gene expression profiles in AILI mice shows that the increased expression of HGF, EGF, VEGF, IL-6, and TNF-α is associated with extensive inflammation and liver injury [246]. Studies of excess APAP-stimulated mouse and human primary hepatocytes have found very rapid, intense, and sustained activation of EGF receptor (EGFR) in the early stage of injury, which are possibly triggered by GSH depletion, after which levels of a series of toxicity markers begin to rise, and activation of EGFR signaling may contribute to late liver regeneration [247]. Viswanathan et al. detected chemokines in a conditioned medium of AILI-derived hepatocytes revealing the higher abundance of VEGF-D (11-fold), VEGF (1.7-fold), its receptors VEGF-R3 and VEGF-R2 (sixfold), and lower expression of VEGF-R1 (0.7-fold) and HGF-R (0.6-fold), and VEGF may activate the STAT3 and ATM pathways[186]. Bone-Larson et al. demonstrated that CXCL10 protects MIP-2 receptors against APAP hepatotoxicity by inducing HGF production [235]. Thus, it is evident that multiple growth factors are involved in the pathological process of AILI.

### Chemokine family

Chemokines act as immunomodulators of drug hepatotoxicity, working cooperatively with cytokines and immune cells locally, whether they end up promoting hepatotoxicity or hepatoprotection depends on various factors, including the type of chemokines, their receptors, target cells, and drugs. Chemokines released from the injured site play a key role in attracting inflammatory cells during the inflammatory response. However, the exact role of these chemokines is still controversial.

CCR2-deficient mice have enhanced sensitivity to APAP, which is associated with increased expression of TNF-α and IFN-γ in the liver, and chemokines contribute to liver regeneration by improving inflammation resolution through M2 macrophage polarization [246, 248, 249]. Several chemokines produced in the liver, such as CCL2, CXCL2, and CXCL10 exert hepatoprotective effects in AILI by acting on their receptors CCR2, CXCR2, and CXCR3, respectively [246]. However, these results contradict some previous reports, in which CCR2 and CCL2-deficient mice do not show a protective effect [116, 250]. In addition, bone marrow-derived monocytes expressing the CCR2 receptor are summoned by CCL2 to the liver and necrotic areas, leading to aggravation of APAP hepatotoxicity [70]. Pharmacological inhibition of CCL2 or CCR2 may have therapeutic potential by reducing the early inflammatory response [251]. These results suggest a pro-inflammatory role of these chemokines in APAP hepatotoxicity. A recent study showed that in the AILI mouse model, levels of RANTES (CCL5) and Eotaxin (CCL11) decreased, while levels of CXCL3, CXCL13, CXCL15 and CXCL16 increased [186]. APAP increases CCL24 in an IL-33 and macrophage-dependent manner [33]. Mice in APAP challenge are found to exhibit severe systemic inflammation and lung injury, and blocking neutrophils infiltration by anti-granulocyte receptor 1 depletion or combined with CXCR2-formyl peptide receptor 1 antagonism significantly prevent APAP-induced hepatotoxicity and associated organ injury[252]. CXCL1 and CXCL2 released by KCs are confirmed to be pro-inflammatory chemokines which take responsible to disease exacerbation [51]. Transcriptome analysis showed that expression of pro-inflammatory genes is enhanced in the liver of mice overexpressing CCL7^tgIEC^, and TLR2 shows a pro-inflammatory pattern of immune dysregulation, which is associated with accelerated progression of AILI [253]. Chen et al. found that APAP significantly upregulates CXCL2, CCL2, CXCR1, CXCR2 and CCR2 levels, and neutrophils recruitment to the liver is dependent on CXCL2 and CCL2 [230]. Moreover, excessive APAP increases hepatic CXCL16 levels, CXCL16 deficiency significantly reduces the production of pro-inflammatory cytokines TNF-α and IL-6, and inhibits neutrophils migration to the site of injury, other chemokines CXCL1 and CXCL2 expression are also diminished, suggesting that CXCL16 may be involved in neutrophil infiltration in APAP hepatotoxicity [38]. APAP-induced increased CD11b^+^/Ly6C^hi^ macrophages is reduced in galectin-3 knockout mice, which is related to decreased expression of chemokines CCL2, CCL3 and their receptors CCR1 and CCR2 [254]. A study of the inflammatory response during liver-lung interaction at the onset of APAP-induced toxicity shows that significantly elevated levels of CCL11 and IL-12 are detectable in the lung, and immunological neutralization of CCL11 improves the appearance of lung tissue in APAP-stimulated mice [255]. Changes in the expression of different chemokines are found in studies of various interventions to alleviate AILI [72, 176, 177, 207, 208, 256, 257], and the mechanism of each chemokine in AILI needs further investigation.

Increasing levels of IL-3 and IL-8, which belong to the chemokine family, are also associated with AILI [208, 258–261]. APAP exposure significantly induces TLR4 expression and promotes increased expression of IL-8, TNF-α, IL-1β and IL-10RA, and combined lipopolysaccharide/APAP exposure induces human primary hepatocytes and KCs to produce the pro-inflammatory cytokine IL-8, but inhibits IL-6 secretion with increasing doses of APAP [259]. Gastrin-releasing peptide receptor (GRPR) antagonists impair IL-8-targeting neutrophils recruitment in vitro, inhibit CXCL2-induced chemotaxis and reduce neutrophils activation by modulating CD11b and CD62L in vivo [258]. Taken together, numerous studies have confirmed the importance of chemokines in APAP-induced hepatotoxicity and repair progression, which may provide hope for specific treatment of the disease.

### Inflammasome

Inflammasomes are multi-protein complexes that contain a receptor, an adapter and an effector. APAP-stimulated hepatocyte necrosis releases cell contents that namely DAMPs, which bind to PRRs and activate inflammasome to trigger an inflammatory cascade response. Inflammasomes are polyprotein oligomers contributing to the inflammatory response and are key mediators in increasing the release of pro-inflammatory cytokines. Several studies confirmed that inflammasome plays an essential role in APAP-induced hepatotoxicity. Overdose APAP increases NF-κB (p-p65) phosphorylation and caspase-1 cleavage, a marker of inflammasome activation [89]. Studies suggest that APAP increases the expression levels of NLRP3 and TLR9, and the formation of the NLRP3 inflammasome is directly attributed to the late toxicity of APAP [262]. Pretreatment with allicin inhibits activation of NLRP3 signaling, and significantly reduces caspase-1 cleavage and IL-1β production [262]. It was reported that IL-22 ameliorates the inflammatory response by downregulating APAP-induced NLRP3 inflammasome activation and releasing mature IL-1β in damaged tissue [201]. Kaempferol alleviates AILI by inhibiting HMGB1/TLR4/NF-κB signaling pathway and NLRP3 activation [263]. Emodin protects hepatocytes against APAP-induced injury by upregulating the nuclear factor erythroid2-related factor 2 (NRF2)-mediated antioxidant stress pathway, inhibiting NLRP3 and downregulating the cGAS-STING signaling pathway [239]. Activation of NLRP3 inflammasome is responsible for providing the second signal required for IL-1β activity in APAP hepatotoxicity. The interaction between necroptosis and NLRP3 inflammasome plays a crucial role in promoting AILI [264]. However, another study found that the symptoms of AILI in mice absence of NLRP3 inflammasome components (ASC^−/−^, NLRP3^−/−^, caspase1^−/−^) are not significantly different from WT mice. Treatment with aspirin, which attenuates the activation of NLRP3 inflammasome, does not reduce the release of DAMPs, accumulation of hepatic neutrophils and liver injury. Thus, they consider the activation of NLRP3 inflammasome may have little effect on APAP hepatotoxicity [265]. Moreover, a study in a piglet model showed that APAP reduces mRNA expression of NLRP3 inflammasome, IL-1 β and IL-18 [266]. The reasons for the opposite results may be the use of different animal models. Whether pharmacological inhibition of NLRP3 inflammasome is a credible strategy for the treatment of AILI remains further investigation.

## Immune-based biomarkers and immune interventions in AILI

Traditionally, the diagnosis of AILI always depends on accurately tracing the medication history and testing liver biochemical levels, including ALT, AST, TBIL, etc. [7] Although such traditional biomarkers are useful to reflect the levels of liver injuries, they are limited in their ability to distinguish DILI from other liver damages and response prognosis, which making them not ideal as biomarkers. The development of biomarkers provides new strategies for the prognosis prediction and targeted therapeutic intervention in AILI. Several promising biomarkers have been identified from APAP-overdose research, such as elevated levels of acetylation of the DAMP molecule HMGB1, which correlates with poor prognosis and outcome [267]. Increased circulating CSF-1 is considered as a biomarker for liver regeneration and improved prognosis [268]. Moreover, its receptor MCSFR has been found to be the most promising candidate marker for predicting the death/transplantation prognosis in DILI, and may help to differentiate between predictive and idiosyncratic DILI [269]. Osteopontin, secreted by a variety of cells, is the molecule that links inflammation and liver recovery/regeneration and, in addition to being an extracellular matrix protein is also a pro-inflammatory cytokine and is considered to have the greatest prognostic value in predicting liver transplantation in patients with DILI compared to other promising biomarkers such as ccK18/K18 and MCSFR [269]. CCL2 (MCP1) is responsible for the initial recruitment of monocytes and other immune cells under inflammatory conditions, and its levels are positively correlated with the degree of liver injury and negatively correlated with the number of circulating monocytes, whose prognostic potential is of high assessment value [270]. Steuerwald et al. used four low-expressing immune complexes (IL-9, IL -17, PDGF-bb, RANTES) and albumin to develop a model that could predict death rate within 6 months of the onset of DILI, with an accuracy of 96% in predicting early death from DILI [271]. In addition, the extent of infiltration of neutrophil- and monocyte-derived macrophages at the site of liver injury, the balance between M1- and M2-type macrophages, the amount of pro-inflammatory Ly6C^hi^CX3CR1^lo^ to reparative Ly6C^lo^CX3CR1^hi^ macrophage transformation, and the number of DC64L^low^DC44^hi^CD4^+^ T cells in the liver in the early stages of APAP hepatotoxicity might serve as predictors of the extent of liver injury and inflammation in APAP hepatotoxicity. However, most of these biomarkers derived from immune cells or inflammatory mediators are still in the realm of preclinical models and knowledge of their practical use in the clinic is very limited, their validity, specificity and sensitivity need confirmation, especially in comparison to the traditional ones.

Currently, the only specific antidote for AILI remains NAC, but the benefit of NAC tends to decrease with the time passed between APAP challenge and treatment. Immune intervention seems to be important, due to the crucial role of immune response in AILI. As mentioned above, there has been considerable literature demonstrating the beneficial effects of targeted interventions on specific immune cell types and specific inflammatory mediators in the treatment of APAP-induced hepatotoxicity. Potential therapeutic targets for immune cells and inflammatory mediators in AILI include: (1) Macrophages activation and exchange are a dynamic process during APAP damage to liver innate immunity, and phenotypic conversion of inflammatory macrophages (e.g. Ly6C^hi^) to reparative (Ly6C^low^) macrophages by promotion or injection of replacement activated macrophages is expected as a potential treatment for AILI, and clinical grade autologous human monocyte-derived macrophages have been reported to be safe in patients with cirrhosis [72]. (2) Excessive neutrophils infiltration leads to severe inflammation and necrosis of liver tissue, and by regulating neutrophils recruitment and activation may be new promising strategies for the treatment of APAP hepatotoxicity, and studies have shown that the use of neutrophil elastase inhibitors to treat animals with overdose APAP hepatotoxicity is comparable to NAC [76]. (3) APAP hepatotoxicity might be treated by blocking the expression of Fas ligands on natural killer cells, Kupffer cells and other cells that promote apoptosis and the persistence of inflammation [272]. (4) Treg cells are recruited by CXCR3 axially in the liver and secrete the anti-inflammatory mediators IL-10 and TGF-β. Regulation of the balance between Th1/Treg cells may be a potential target for the treatment of AILI. (5) Eosinophil-based cellular therapeutic approaches may be one of the potential therapeutic targets for AILI, and their positive role in AILI warrants further investigation. (6) Targeted drug delivery to modulate the expression of inflammatory mediators may be a new approach to treat APAP hepatotoxicity. Both using receptor antagonists or chemical inhibitors to reduce the expression of pro-inflammatory cytokines such as IL-1, IL-17, IL-18, IL-23, IFN-γ, TGF-β, CXCL16, NLRP3, and by in vitro injection or chemical intervention to increase the expression of anti-inflammatory cytokines, including IL-10, IL-13, IL-15, IL-22, IL-24, IL-33c, CCR2, CXCL10, G-CSF, EGF, VEGF, can help to reduce liver inflammation and promote liver damage repair. (7) By modulating the expression of chemokines that enhance local responses in the liver, it may offer hope for specific treatment of drug hepatotoxicity. Scientists found that CSF-1-targeted repair of innate macrophages function can successfully promote liver regeneration after toxic exposure [268]. (8) HMGB1, the promoter of the immune response in the AILI process, is also a potential therapeutic target for APAP hepatotoxicity.

## Conclusion and perspectives

The mechanisms underlying AILI are intricate, ranging from hepatocyte necrosis to aseptic inflammation to liver regeneration, and are related to various intracellular and extracellular events. Researches using intervention by analyzing the role of inflammatory mediators in AILI are summarized in Table 1. The objective of this current review is to critically summarize the immunological mechanisms of APAP-induced hepatotoxicity, with emphasis to clarify the roles of immune cells, cytokines, chemokines, and inflammasome in the development of AILI, a better understanding of these inflammatory mechanisms offers hope for the discovery of new therapeutic targets, especially for the transition from the injury to the regenerative phase (Figs. 1 and 2).

**Table 1:** Role of inflammatory mediators in acetaminophen induced live injury

Cytokines category	Name	Intervention means	Performance	Ending	Refs
IL-1 family	IL-1R	IL-1R deficient mice or antagonists	Decrease	Attenuate inflammatory response	[55, 179]
	IL-18	Treatment with benzyl alcohol and TLR4 knockdown mice	Decrease	Protective role	[180]
		IL-18 binding protein	Decrease	Protective role	[181]
	IL-33	IL-33 deficient mice	Decrease	Aggravate inflammatory response	[33, 183]
		Blocking IL-33/IL1RL1 axis	Decrease	Attenuate APAP-mediated organ injury	[74]
	IL-36γ	IL-36 receptor antagonist	Decrease	Aggravate liver injury	[184]
IL-2 family	IL-4	IL-4 deficient mice	Decrease	Protective role	[188]
	IL-13	IL-13 deficient mice	Decrease	Protective role	[187]
	IL-15	IL-15 knockout mice	Decrease	Increase susceptibility	[190]
IL-6 family	IL-6	IL-6 deficient mice	Decrease	Increase susceptibility	[193]
IL-10 family	IL-10	IL-10 knockdown mice	Decrease	Increase susceptibility	[197, 198]
		SOCS2 knockout mice	Decrease	Enlarge injury site	[200]
	IL-22	In vitro prophylactic injection of IL-22	Increase	Promote hepatocyte proliferation	[174]
		IL-22 binding protein	Decrease	Improve inflammatory response	[202]
IL-17 family	IL-17	IL-17 knockdown mice	Decrease	Attenuate hepatotoxicity	[159]
Other interleukin cytokines	IL-11	Deleting IL-11RA1 in hepatocytes/knockdown IL-11 in mice	Decrease	Inhibit hepatocyte death	[215]
	rhIL-11	In vitro injection	Increase	Protective role	[217]
TNF	TNF-α	TNF-α or TNF- R1 deficient mice	Decrease	Attenuate hepatotoxicity	[219–223]
	TNF-α, TGF-β	NTPDase2 deficient mice	Increase	Aggravate inflammatory response	[232]
Interferon	IFN-1	Interferon α/β receptor deficient mice	Decrease	Delay APAP-mediated liver injury	[236]
		IFN-1R deficient mice	Decrease	Express iNOS impair	[237]
	IFN-γ	IFN-γ deficient mice	Decrease	Reduce susceptibility	[111]
		5-lipoxygenase knockout mice	Decrease	Attenuate liver disease	[233]
		MIF knockout mice	Decrease	Attenuate liver disease	[234]
		Pretreatment anti-IFN-γ antibody in CCR2 deficient mice	Decrease	Attenuate liver disease	[111]
Growth factor	TGF-β1	GW788388	Decrease	Ameliorates hepatocyte inflammation	[245]
Chemokine family	IL-8	GRPR antagonists	Decrease	Reduce neutrophil activation	[258]
	CCR2	CCR2 deficient mice	Decrease	Increase susceptibility	[246, 248]
	CXCL16	CXCL16 deficiency mice	Decrease	Reduce neutrophil infiltration	[38]
	CCL2, CCL3	Galectin-3 knockout mice	Decrease	Reduce CD11b^+^/Ly6C^hi^ macrophages	[254]
Inflammasome	NALP3	ASC/ NALP3/caspase1 deficient	Decrease	Disease manifestations were not significantly change	[265]

As mentioned above, several immune cells and inflammatory mediators seem to have a dual role in aggravating liver damage and promoting liver repair and regeneration, which, to some extent, affects scientists to translate this knowledge into effective treatment of AILI (with NAC being still the only FDA approved antidote). These contrary results may be partly attributed to the opposite inflammatory activities produced by different immune components through various crosstalk signals. Moreover, some immune interventions have a high probability of untargeted effects, and the multiple targets of drugs also lead to controversial experimental results. Meanwhile, the experimental setting and the exposure level of APAP have an important impact on the research results. For example, different vehicles (phosphate buffered saline, stroke-physiological saline solution) and different administration routes (intraperitoneal injection, intravenous injection, oral administration, intragastric administration), leading to varying degrees of liver damage. Different strains or even mice of the same strain from different suppliers may have diverse immune response, leading to the opposite outcome. Besides, the dosage range of APAP in different kinds of experimental animals is not exactly the same, diverging levels of APAP exposure may initiate varying degrees of liver immune response. Therefore, rather than simply comparing the current results, the whole literature and experimental setting should be considered when controversial results occur. Although there is great progress in DILI treatment, there is still lack of high-quality data support from strictly prospective controlled studies, and is far from the application of standardized norms. More detailed research in this field and critical discussion of the current controversial conclusions are urgently needed in the future. The rigor of experimental design, the complex interactions of immune responses, off-target effects of reagents and genetic interventions on inflammation, as well as the role of inflammatory mediators in AILI should be considered. All aspects of the immune response pathophysiology should be considered in the experimental design, which help to clarify the potential role of the liver immune system in aggravating tissue damage or promoting regeneration. An accurate and detailed understanding of the activation of the immune system and the sequence of events mediated by inflammatory mediators leading to liver injury could provide potential therapeutic strategies for APAP hepatotoxicity. Overall, the initiative to understand the role of immune cells and inflammatory mediators in AILI is expected to provide an innovative, viable approach for clinical treatment.

## Data Availability

Not applicable.

## Abbreviations

APAP: Acetaminophen
AILI: APAP-induced liver injury
DILI: Drug-induced liver injury
HDS: Herbals and dietary supplements
ALF: Acute liver failure
NAPQI: N-acetyl-p-benzoquinone imine
GSH: Glutathione
DAMPs: Damage-associated molecular patterns
NAC: n-acetylcysteine
KCs: Kupffer cells
MoMF: Monocyte-derived macrophages
Mincle: Macrophage-inducible C-type lectin
SAP130: Spliceosome-associated protein 130
WT: Wild-type
HSCs: Hepatic stellate cells
TNF: Tumor necrosis factor
IL: Interleukin
ROS: Reactive oxygen species
RNS: Reactive nitrogen species
C1q: Complement 1q
LSECs: Liver sinusoidal endothelial cells
Mrp4: Multidrug resistance-associated protein 4
HMGB1: High-mobility group box1
OPN: Osteopontin
CXCL: C–X–C motif chemokine ligand
NE: Neutrophil elastase
miR-233: MicroRNA-223
TLR9: Toll-like receptor 9
CLEC-2: C-type lectin-like receptor-2
NK: Natural killer
NKT: Natural killer T
IFN: Interferon
SJL: Swiss Jim lambert
DMSO: Dimethyl sulfoxide
DCs: Dendritic cells
mDCs: Myeloid dendritic cells
pDCs: Plasmacytoid dendritic cells
MHC: Major histocompatibility complex
CCl_4_: Carbon tetrachloride
CCL: C–C motif chemokine ligand
TGF: Transforming growth factor
ILCs: Innate lymphoid cells
PRRs: Pattern recognition receptors
ICAM-1: Intercellular adhesion molecule-1
VCAM-1: Vascular cell adhesion molecule-1
MIP: Macrophage inflammatory protein
HSPs: Heat shock proteins
NF: Nuclear factor
iNOS: Inducible nitric oxide synthesis
SOCS: Suppressor of cytokine signaling
NLRP3: NOD-like receptor thermal protein domain associated protein 3
IL-22BP: IL-22 binding protein
LC3-II: PE-conjugated form of light chain 3
p-AMPK: Phosphorylation of AMP-activated kinase
CSF: Colony stimulating factor
M-CSF: Macrophage-CSF
G-CSF: Granulocyte-CSF
GM-CSF: Granulocyte–macrophage-CSF
IL-1RA: IL-1 receptor antagonist
ERK: Extracellular regulated protein kinases
STAT3: Signal transducer and activator of transcription 3
PRHs: Primary rat hepatocytes
rhIL-11: Recombinant human IL-11
TNFsR: Tumor necrosis factor receptor
sDR5-Fc: Soluble death receptor 5-Fc fusion protein
TRAIL: TNF-related apoptosis-inducing ligand
PDGF: Platelet-derived growth factor
IRF-1: Interferon regulatory factor-1
HSC-CM: HSCs-derived paracrine factor
cGAS: Cyclic GMP-AMP synthase
EGF: Endothelial growth factor
VEGF: Vascular endothelial growth factor
SERPINB: Serine protease inhibitor family B
HGF: Hepatocyte growth factor
CCR: C–C motif chemokine receptor
CXCR: C–X–C chemokine receptor
GRPR: Gastrin-releasing peptide receptor
NRF2: Nuclear factor erythroid2-related factor 2
